# Errata

**Published:** 2014-12-12

## Vol. 63, No. RR-5

In the MMWR Recommendations and Reports “Human Papillomavirus Vaccination: Recommendations of the Advisory Committee on Immunization Practices (ACIP),” an error occurred on page 5. The last sentence of the first full paragraph should read, “Among these six cancers, approximately **21,300** were attributable to HPV16/18 (**7,900 [37%]** among men and **13,400 [63%]** among women) (Table 2).”

In Table 2 on page 6, the average annual numbers of cancers attributable to HPV 16/18 were incorrect. Following is the corrected table:

**TABLE 2:** Average annual number and percentage of cancer cases attributable to human papillomavirus and to HPV 16 and HPV 18, by anatomic site and sex — United States, 2006–2010.

Anatomic site	Average no. of cancers per year in sites where HPV is often found (HPV-associated cancers)*			Cancers attributable to any HPV				Cancers attributable to HPV 16/18			
		
	Male	Female	Both sexes	%	Average no.†			%	Average no.†		
	
					Male	Female	Both sexes		Male	Female	Both sexes
Cervix	0	11,422	11,422	91§	0	10,400	10,400	67	0	**7,700**	**7,700**
Anus	1,549	2,821	4,370	91	1,400	2,600	4,000	**79**	**1,200**	**2,200**	**3,400**
Oropharynx	9,974	2,443	12,417	72	7,200	1,800	9,000	**62**	**6,200**	**1,500**	**7,700**
Penis	1,048	0	1,048	63	700	0	700	48	**500**	0	**500**
Vagina	0	735	735	75	0	600	600	**57**	0	**400**	**400**
Vulva	0	3,168	3,168	69	0	2,200	2,200	49	0	**1,600**	**1,600**
Total	12,571	20,589	33,160		9,300	17,600	26,900		**7,900**	**13,400**	**21,300**

**Abbreviation:** HPV = human papillomavirus.

* **Sources:** Data come from population-based cancer registries that participate in the National Program of Cancer Registries and/or the Surveillance, Epidemiology, and End Results Program, and meet criteria for high data quality. Cancer Registry Data are from all states meeting USCS publication criteria (http://www.cdc.gov/cancer/npcr/uscs/technical_notes/criteria.htm) for all years 2006–2010 and cover approximately 94.8% of the US population. In order to determine those cancers most likely to be HPV-associated, the following additional criteria were applied to the NPCR/SEER data: all cancers were microscopically confirmed**; c**ervical cancers were limited by histology to carcinomas only (ICD-O-3 histology codes 8010–8671, 8940–8941)**; a**ll other cancer sites were limited by histology to squamous cell carcinomas only (ICD-O-3 histology codes 8050–8084,8120–8131)**; o**ropharyngeal cancers were defined as having the following ICD-O-3 site codes: 19, 24, 28, 90–91, 98–99, 102, 108–109, 140, 142, and 148.

† The estimated number of HPV-attributable or HPV 16/18-attributable cancers was calculated by multiplying the HPV-associated cancer counts by the percentage of each cancer attributable to HPV or HPV16/18. Estimates rounded to the nearest 100.

§ Although HPV is accepted to be a necessary factor in the causal pathway to invasive cervical cancer, HPV is not always detected in tumor specimens from women who receive a diagnosis of invasive cervical cancer due to a variety of reasons, including misclassification of tissue specimens as cervix, quality of tissue specimens, assay sensitivity, and a small proportion of HPV-negative, cervical cancers.

